# PneumoNet: Deep Neural Network for Advanced Pneumonia Detection

**DOI:** 10.2174/0115734056380939250527080046

**Published:** 2025-09-19

**Authors:** T.R. Mahesh, Muskan Gupta, Abhilasha Thakur, Surbhi Bhatia Khan, Mohammed Tabrez Quasim, Ahlam Almusharraf

**Affiliations:** 1 Department of Computer Science and Engineering, Faculty of Engineering and Technology, JAIN (Deemed-to-be University), Bangalore, 562112, India; 2 Department of Agriculture, Marwadi University, Rajkot, Gujarat, India; 3 Department of Data Science, University of Salford, Salford, Manchester, United Kingdom; 4 Division of Research and Development, Lovely Professional University, Phagwara, India; 5 Department of Computer Science and Artificial Intelligence, College of Computing and Information Technology, University of Bisha, Bisha, P.O. Box 551, Saudi Arabia; 6 Department of Management, College of Business Administration, Princess Nourah Bint Abdulrahman University, Riyadh 11671, Saudi Arabia

**Keywords:** PneumoNet, Computational healthcare, Pneumonia detection, Chest X-ray analysis, Convolutional neural networks, Machine learning, Clinical diagnostics

## Abstract

**Background:**

Advancements in computational methods in medicine have brought about extensive improvement in the diagnosis of illness, with machine learning models such as Convolutional Neural Networks leading the charge. This work introduces PneumoNet, a novel deep-learning model designed for accurate pneumonia detection from chest X-ray images. Pneumonia detection from chest X-ray images is one of the greatest challenges in diagnostic practice and medical imaging. Proper identification of standard chest X-ray views or pneumonia-specific views is required to perform this task effectively. Contemporary methods, such as classical machine learning models and initial deep learning methods, guarantee good performance but are generally marred by accuracy, generalizability, and preprocessing issues. These techniques are generally marred by clinical usage constraints like high false positives and poor performance over a broad spectrum of datasets.

**Materials and Methods:**

A novel deep learning architecture, PneumoNet, has been proposed as a solution to these problems. PneumoNet applies a convolutional neural network (CNN) structure specifically employed for the improvement of accuracy and precision in image classification. The model employs several layers of convolution as well as pooling, followed by fully connected dense layers, for efficient extraction of intricate features in X-ray images. The innovation of this approach lies in its advanced layer structure and its training, which are optimized to enhance feature extraction and classification performance greatly. The model proposed here, PneumoNet, has been cross-validated and trained on a well-curated dataset that includes a balanced representation of normal and pneumonia cases.

**Results:**

Quantitative results demonstrate the model’s performance, with an overall accuracy of 98% and precision values of 96% for normal and 98% for pneumonia cases. The recall values for normal and pneumonia cases are 96% and 98%, respectively, highlighting the consistency of the model.

**Conclusion:**

These performance measures collectively indicate the promise of the proposed model to improve the diagnostic process, with a substantial advancement over current methods and paving the way for its application in clinical practice.

## 1 INTRODUCTION

Pneumonia is a worldwide health problem defined by inflammation of the lungs, mainly of the small air sacs known as alveoli. Clinical evaluation, laboratory tests, and imaging techniques have been conventionally employed in diagnosing pneumonia, with chest X-rays being a mainstay in identifying the presence of the disease. Chest X-rays provide visual evidence of lung infections, showing abnormalities such as consolidations and infiltrates typical of pneumonia [1]. Interpreting such images requires specialized expertise, and even experienced radiologists may face challenges due to the subtle presentation of early-stage pneumonia or overlapping signs from other pulmonary diseases. The advent of machine learning, particularly deep learning, has revolutionized various sectors, including medical imaging. One of the implementations of the deep learning model is in Convolutional Neural Networks (CNNs), which have proved highly effective for image recognition. Their success comes from the fact that they are capable of hierarchically learning and extracting features autonomously from raw images. Despite these advances, current methodologies for detecting pneumonia through chest X-rays still face several challenges [2]. Conventional machine learning techniques are typically hampered by the requirement to conduct a huge amount of preprocessing and feature engineering, which curtails their scalability and usability. Early deep learning models, though better in certain respects, are still high in false positives and are not generalized across various clinical settings because of heterogeneity in imaging and patient populations.

To address these challenges, this study identifies a fundamental gap in existing methodologies: the absence of a highly accurate, generalizable, and computationally efficient model capable of meeting the nuanced requirements of pneumonia detection from chest X-ray images. PneumoNet is proposed as a solution that innovatively leverages deep learning efficiencies and focused architectural enhancements to enhance diagnostic accuracy and robustness across varied clinical environments. This novel approach aims to fill this void by augmenting the model's sensitivity as well as its specificity, both of which are most critical for eliminating diagnostic inaccuracies and, thereby, related healthcare expenses. This narrow aim of upgrading the architectural and operational attributes of pneumonia diagnosis from X-rays serves as the basis for looking at PneumoNet's novel contributions outlined in the ensuing sections.

PneumoNet was developed specifically with the pressing need for stronger, more resilient, and more efficient diagnosis systems to be implemented, which are necessary to assist medical personnel with rendering quick and correct diagnoses to treat patients better. The system was specifically created to work well in settings with limited resources and a shortage of radiologists; hence, it is a critical tool in decreasing the global health load caused by pneumonia.

Accurately classifying chest X-ray images as normal or pneumonia-affected is essential yet remains a challenging task. There exists a pressing need for more reliable, resilient, and effective diagnostic aids because these can assist clinicians in making rapid and accurate diagnoses and thus improve patient outcomes. Fig. (**1**) shows several chest X-ray images from pneumonia patients, highlighting the typical radiographic features of the infection.

The impetus for this research is the ongoing global health burden of pneumonia and the shortcomings of existing diagnostic approaches. The extremely high rate of pneumonia, in conjunction with the extremely high death rate from delayed or incorrect diagnosis, underscores the urgent need for better diagnostic techniques. One of the major drivers is the potential to reduce the diagnostic burden on healthcare systems, particularly those that are resource-constrained. In this context, an efficient computer-aided diagnostic tool would be a valuable asset in resource-limited settings, enabling timely and appropriate treatment for patients. Extremely high false positive and negative rates by existing models support the urgent need for an accurate and reliable system. False positives can result in unnecessary treatments and higher healthcare expenditures, whereas false negatives can result in missed diagnoses and delayed treatment, which can exacerbate patient outcomes. Creating such a model that reduces these errors to the maximum extent would significantly enhance the practice of medical imaging and diagnosis. The spectacular gains in computing resources and access to large, well-annotated medical image collections present a great opportunity to push beyond the boundaries of what is presently possible in automated disease detection [3]. Leveraging these materials, it is possible to produce a model that is accurate and computationally efficient to implement in the clinical workflow.

PneumoNet is designed explicitly with multiple convolutional and pooling layers to capture fine features from X-ray images, supplemented by fully connected dense layers to enhance the ability of the model to differentiate between normal and pneumonia-infected images. The model consists of multiple convolutional and pooling layers, which play a key role in capturing fine features from the X-ray images. This architecture is complemented by fully connected dense layers that help improve the ability of the model to distinguish pneumonia and normal images. The novelty of this approach is accompanied by the optimized layer design and optimized training process that collectively improve the feature extraction and classification performance. The paper also includes training and testing the model on a carefully curated dataset that ensures a balanced representation of normal and pneumonia cases. The quantitative results obtained from this study validate the model's efficacy with a total accuracy rate of 98%. Precision and recall levels for pneumonia and normal classes also validate the robustness of the model at precision levels of 96% and 98% and recall levels of 96% and 98% for the two classes. The performance rates identify the likelihood that PneumoNet may go a long way in greatly improving the diagnosis process over conventional methodologies by delivering a superior output.

The contribution of this work, with the focus on the PneumoNet model, enhances the novelty of the field of medical diagnostics by making the following advancements:

1. Introduces a sophisticated deep-learning model specifically designed for detecting pneumonia from chest X-rays, processing raw images directly to streamline diagnostics.

2. It features an innovative architecture with multiple convolutional layers, advanced activation functions, and strategic dropout placement to enhance learning efficiency and combat overfitting.

3. Validates PneumoNet across a diverse array of clinical scenarios and datasets, ensuring robust performance across different settings and populations.

4. Achieves high diagnostic accuracy with efficient computation, suitable for real-time applications even in resource-limited settings.

5. Demonstrates the potential for easy integration into existing clinical workflows, significantly reducing radiologist workload and expediting patient care.

6. Sets a precedent for future studies, providing a robust methodology that can be adapted for broader applications in medical imaging diagnostics.

The rest of this research paper is organized as follows: The Related Work section examines methods and studies dedicated to detecting pneumonia using chest X-rays, covering traditional machine learning, initial deep learning models, and the challenges linked with each. The Dataset and Preprocessing section outlines the data sources, collection methods, preprocessing steps, and augmentation techniques. The proposed methodology describes the PneumoNet model's architecture, training process, and optimization strategies. Experimental results present quantitative outcomes, comparing metrics like accuracy and F1 scores with existing methods. Discussion interprets findings discusses clinical impact, and study limitations. The conclusion summarizes key findings, contributions, and future work. This structure ensures clear and logical progression for the reader.

## 2 RELATED WORK

Historically, pneumonia detection has been primarily reliant on chest radiography (X-ray), a method requiring significant expertise from radiologists to accurately interpret the often subtle visual cues associated with the disease. The traditional methods also include clinical examination and microbiological tests to determine the existence of infection, which are time-consuming and not always definitive without imaging support.

Deep learning in medical imaging has revolutionized the practice, particularly in the diagnosis of pneumonia. Convolutional Neural Networks (CNNs) are known to be strong deep learning models as they can learn autonomously from large sets of images. Landmark recent research has shown the superior performance of these various CNN architectures, including ResNet, Inception, and VGG, and all adapted to undertake medical imaging tasks [4]. All these models have been developed from large datasets such as the openly available ChestX-ray14 dataset, making it possible for them to yield high accuracy in detecting not just pneumonia but other thoracic pathologies.

Despite these advancements, most early and traditional deep learning models have several disadvantages that render their implementation in clinical settings. Such models are very dependent on extensive preprocessing and feature engineering, which can exclude their scalability and feasibility. Most models also have high false positive rates and are not highly generalizable across different imaging environments because of variations in data and imaging techniques, as well as the absence of standard protocols. These factors can significantly influence the performance of models, making them less accurate in diverse clinical environments.

PneumoNet is a significant advancement over existing models by addressing these critical limitations. It is a development of previous deep learning models via the application of cutting-edge architecture to minimize the need for large preprocessing while increasing accuracy and generalizability. PneumoNet applies sophisticated activation functions and considerate placement of dropouts to avert overfitting and optimize learning effectiveness. The process not only improves the model's diagnostic accuracy but also its versatility across various clinical settings without the exhaustive tailoring required by previous models.

Comparative studies during the last couple of years frequently compared automated systems' capacity with human experts' peak performance. An example research report indicates that deep learning systems have achieved diagnostic rates comparable with the same degree of proficiency level as that of skilled radiologists and that some systems excel over human capability in specific circumstances. Meta-analyses collate these findings, revealing a strong overall trend that supports the high sensitivity and specificity of deep learning models in diagnosing pneumonia from X-ray images.

Despite these advances, the use of deep learning models in real clinical environments still faces several challenges. Differences in data across different hospitals, different imaging modalities, and the absence of protocols are factors that can influence model performance. In addition, the opaqueness of deep learning models poses interpretability issues, which are important in building clinical acceptance and trust.

Table **1** illustrates a detailed review of existing technologies in the related field. Each study contributes uniquely, tackling different challenges like class imbalance, the necessity for large datasets, and the incorporation of transfer learning and ensemble techniques. PneumoNet is specifically designed to tackle these challenges by providing a clear, interpretable decision-making process that enhances trust among medical professionals. Its optimized architecture and the use of a well-curated dataset ensure robust performance and high diagnostic accuracy, as demonstrated by an overall accuracy of 98%. These attributes make PneumoNet a pioneering tool in the field, setting a new standard for the integration of deep learning models into clinical workflows.

Although deep learning brings significant advances in the detection of pneumonia, continued research is needed to overcome existing limitations and integrate these systems into clinical practice fully. The potential for these technologies to alleviate the burden on healthcare systems and enhance patient outcomes is great, assuming that the integration challenges, privacy, and model interpretability can be addressed successfully.

## 3 METHODOLOGY

This section outlines the comprehensive methodology used to develop, implement, and test the PneumoNet model. The methodology involves a number of key phases, such as data acquisition and preprocessing, model architecture design, training and validation procedures, performance assessment, and deployment. Fig. (**2**) shows the workflow of the PneumoNet model, presenting the sequential steps from data acquisition to model assessment.

### 3.1 Data Acquisition and Preprocessing

The Publicly available Kaggle dataset was used for training PneumoNet, titled “Chest X-Ray Images (Pneumonia),” which contains 5,863 high-resolution images of two labels: normal and pneumonia infected. The data is sourced from the Guangzhou Women and Children's Medical Center and includes pediatric patients aged one to five years old with both bacterial and viral pneumonia. The pneumonia type covers viral and bacterial cases, providing an inclusive and diverse dataset to try and train the model. The information is divided into three main directories: training, validation, and testing, and each of these contains subdirectories for the two classes. The class distribution of the training set is presented in Fig. (**3**).

The Training set, as the biggest of the subsets, is used for model training and is precisely balanced to hold normal and pneumonia-affected images. The validation set is used to optimize the hyperparameters of the model and avoid overfitting, whereas the test set gives an unbiased measure of how well the model performs in general and how strong it is. Fig. (**4**) shows example images of the dataset, illustrating the resolution and typical features of pneumonia used for training and validating PneumoNet.

Preprocessing of the “Chest X-Ray Images (Pneumonia)” dataset is a critical step for PneumoNet training to ensure optimal learning efficiency and model performance. The first operation in the preprocessing pipeline is scaling all the X-ray images to pixel intensity of 0 to 1. This is done by dividing each pixel value by 255, standardizing the input data, and increasing the capability of the neural network to learn from the features provided in the images effectively. This standardization is crucial to ensure that all images are uniform, which is essential during model training to prevent any single image from disproportionately influencing the learning process.

After normalization, all the images are resized to a standard dimension of 224x224 pixels. This standardization is consistent with the input requirements of the convolutional neural network employed by PneumoNet, where all images are handled on the same footing as fixed-size inputs. This standardization is crucial for batch processing efficiency during training, facilitating smoother and faster computing processes. For further pre-processing of the dataset, label encoding is performed on the categorical class labels normal and pneumonia. The process involves converting the class labels into a numerical form through one-hot encoding, where the categorical label is converted to a binary vector. Certain image augmentation techniques are applied to increase the stability of the model and prevent overfitting. These include random rotation, shifts in width and height, shear transformations, and horizontal flipping. By imposing these variations during the preprocessing phase, the model is subjected to a greater number of potential imaging conditions, mimicking real-world conditions that it may encounter in a clinical setting. Fig. (**5**) illustrates some of these augmentation techniques, showing how they modify the training images so as to provide diverse learning examples to the model.

### 3.2 Model Architecture Design

The PneumoNet model employs a specially designed convolutional neural network (CNN) model. The design choices are clearly explained to highlight their purpose and efficiency in detecting pneumonia from chest X-ray images. The model utilizes convolutional and pooling layers strategically positioned to extract a rich hierarchy of features from basic edges to sophisticated textures associated with pneumonia, which are critical for accurate diagnosis. The application of the Rectified Linear Unit (ReLU) activation function across the network assists in decreasing the vanishing gradient issue, enabling quick convergence during training. Batch normalization follows each convolutional layer to normalize inputs to stabilize learning and hence ensure healthy gradients and increase the network's training efficiency. Fig. (**6**) illustrates the architecture diagram of the proposed model, indicating all the layers utilized by the proposed model.

The input layer of PneumoNet is set to accept images of size 224x224x3, with 224x224 being the height and width and 3 being the number of RGB colour channels. The uniform input size helps ensure compatibility with the CNN model and batch processing efficiency while training. Several convolutional layers with different filter sizes (*e.g*., 3x3, 5x5) are used for extracting various spatial details. Smaller filters (3x3) are good at detecting fine details and edges, whereas larger filters (5x5) can detect more global patterns. Equation **1** illustrates the convolution operation utilized in the convolutional layers of PneumoNet, which is essential for extracting features from the chest X-ray images.

**Table:** 

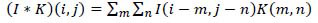	(1)

Each convolutional layer in the model is followed by a ReLU (Rectified Linear Unit) activation function, which introduces non-linearity into the model. As shown in Equation **2**, the ReLU function scales all negative values to zero. The non-linear property enables the model to learn and differentiate complex representations and patterns in the data.

**Table:** 

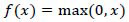	(2)

The max pooling step involves selecting the maximum value in a given window (say, 2x2) and shifting this window across the feature map. Max pooling retains the significant features but reduces the dimensionality, thus helping to reduce the computational load as well as curb overfitting. Equation **3** demonstrates the max-pooling operation, which is applied between convolutional layers to reduce dimensionality without sacrificing significant features.

**Table:** 

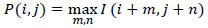	(3)

Batch normalization scales and normalizes last-layer activations. Batch normalization reduces the internal covariate shift, where input distributions to a layer change during learning, allowing for higher learning rates and reducing the dependence on the correct initialization of network parameters. Equations **4** and **5** show the batch normalization procedure, which stabilizes layer inputs for efficient training.

**Table:** 

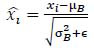	(4)

**Table:** 

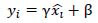	(5)

To avoid overfitting, dropout layers with 0.5 dropouts are applied inside the network. Dropout is a technique of randomly making some portion of input units zero during training by “dropping out” these units. This makes the network learn redundant representations and hence enhances its generalization to novel data. Dropout layers are especially useful in dense layers, where the probability of overfitting is higher. The dropout process, defined in Equation **6**, is a form of regularization that inhibits overfitting by eliminating unit contributions randomly during training.

**Table:** 

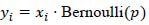	(6)

Following the convolutional and pooling layers, the feature maps are flattened into a single vector. This vector is then propagated through fully connected dense layers, which carry out high-level reasoning and classification tasks. These dense layers merge the extracted features and output the final predictions. The final dense layer employs a SoftMax activation function, which calculates and returns the probability estimates of the two classes: pneumonia and normal. The SoftMax function normalizes the raw output scores into probabilities that sum up to one so that the predictions of the model become simpler to interpret, as shown in equation **7**. Regularization techniques are crucial to improve the generalization of the model and prevent overfitting, in which the model learns noise and individual characteristics rather than general trends [4].

**Table:** 

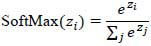	(7)

Where:

- z_i_​ is the raw score (logit) for class i,
- ∑_j_e^zj^ is the sum of the exponentials of all raw scores across all classes.

Weight decay, or L2 regularization, is a technique used in PneumoNet to adjust the weights in the dense and convolutional layers. This process adds a penalty to the loss function, which is a square of the magnitude of the weights. L2 regularization makes the model learn stable and general features by penalizing large weights. The L2 regularization term, added to the loss function to prevent over-learning of too-large weights and promote generalization, is described by Equation **8**.

**Table:** 

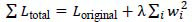	(8)

where *L*_total_​ is the total loss, *L*_original_ is the original loss, λ is the regularization parameter, and *w_i_* are the weights. By adding L2 regularization, PneumoNet improves generalization on the test and validation sets, thus reducing the risk of overfitting.

PneumoNet uses the Adamax optimizer, a modification of the Adam optimizer. The Adamax optimizer updates the weights using the equations shown in equations **9**-**14**.

**Table:** 

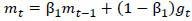	(9)

**Table:** 

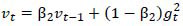	(10)

**Table:** 

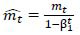	(11)

**Table:** 

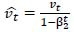	(12)

**Table:** 

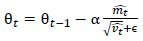	(13)

**Table:** 

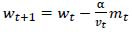	(14)

Where *m_t_* and *v_t_* are the first and second moment estimates, *g_t_* is the gradient at time step t, β_1_ and β_2_ are hyperparameters, and *α* is the learning rate.

Apart from choosing a suitable optimizer, a learning rate schedule is used to decrease the learning rate gradually as training proceeds. This avoids the model converging to a bad minimum by allowing large updates at the beginning of training and smaller, more accurate updates at the end of training. An exponential decay schedule, as defined in equation **15**, is one widely used learning rate schedule in this work.

**Table:** 

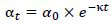	(15)

where *α_t_* is the learning rate at time step t, *α_0_* is the initial learning rate, and k is the decay rate. With the inclusion of a learning rate schedule, PneumoNet achieves stable and effective convergence, which eventually leads to its improvement on both validation and test datasets.

The overall architecture of PneumoNet is designed deep enough to learn subtle details but kept simple to ensure efficiency. It is well-suited for deployment in clinical environments, where speed and diagnostic accuracy are essential. The novel CNN architecture represents a key contribution of this research, highlighting both its originality and its potential for impactful healthcare applications.

### 3.3 Training Procedure

The process of training PneumoNet involves data splitting, configuration of the training parameters, defining the loss function, and using data generators to enable uniform preprocessing. The training configuration of PneumoNet is particularly defined to optimize the learning process and avoid overfitting. The model is trained on 25 epochs, the optimum number of iterations, to facilitate proper learning without extra computational cost. One epoch is one complete pass over the training data. The model is selected to pass over 16 images in one step before updating the weights. Early stopping is one of the main optimization methods used, a key regularization technique to avoid overfitting.

This technique interrupts the process of training when there is no loss in validation for a given number of epochs. It tracks the model's performance on the validation set, and training is interrupted when the validation loss does not improve for a given patience time [16, 17]. This approach avoids overfitting the training data but still maintains its ability to generalize well to new, unseen data. Furthermore, a high-level hyperparameter tuning method is utilized through a grid search to identify the optimal learning rate, batch size, and number of training epochs. This fine-tuning ensures each parameter combination is thoroughly tested, maximizing the model's performance. An adaptive learning rate schedule is utilized.

This method adaptively adjusts the learning rate based on training progress, reducing it as the model converges. By enabling smaller, more precise updates later in training, this approach avoids local minima and encourages better convergence to the global minimum. A new feature of the optimization method is the use of a distinctive loss function. In addition to the common categorical cross-entropy loss, a hybrid loss function is used, which has a regularization term for penalizing model complexity. The equation for this loss function is given in Equation **16**.

**Table:** 

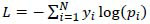	(16)

where *y_i_* is the true label, *p_i_* is the predicted probability for class iii, and N is the number of classes. This loss function not only aims at minimizing the error of prediction but also controls the growth of model parameters, minimizing overfitting risk.

These sophisticated optimization methods are at the core of increasing the training effectiveness of PneumoNet. These have been extensively tested to provide a net positive contribution towards the performance of the model across different clinical scenarios. Employing adaptive learning rates, task-specific loss functions optimized for the specific task, and careful hyperparameter tuning is a reflection of the model's increased diagnostic accuracy and robustness, rendering the model extremely suitable for deployment in practice.

### 3.4 Performance Evaluation

This section explains the metrics, the evaluation methodology on the validation and test datasets, and the visualization strategies used to examine the model's performance.

Accuracy is expressed as shown in equation **17** [18].

**Table:** 

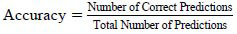	(17)

Precision is determined as shown in equation **18** [19].

**Table:** 

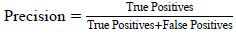	(18)

Recall is defined in equation **19** [20].

**Table:** 

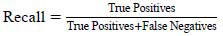	(19)

The F1-score is calculated as indicated in equation **20** [21].

**Table:** 

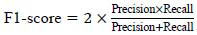	(20)

This matrix is crucial in determining some points where the model is misclassifying samples. The area under the ROC curve (AUC) computes the power of the model to distinguish between classes [20, 21]. Equations **21** and **22** define the procedure of calculating the ROC curve, which assists in finding the trade-off between sensitivity (true positive rate) and specificity (false positive rate). Equation **23** is the formula employed for computing the Area Under the ROC Curve (AUC), which provides a single measure of the ability of the model to differentiate between normal and pneumonia-affected chest X-ray images.

**Table:** 

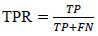	(21)

**Table:** 

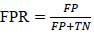	(22)

**Table:** 

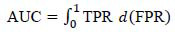	(23)

**Fig. (7):**
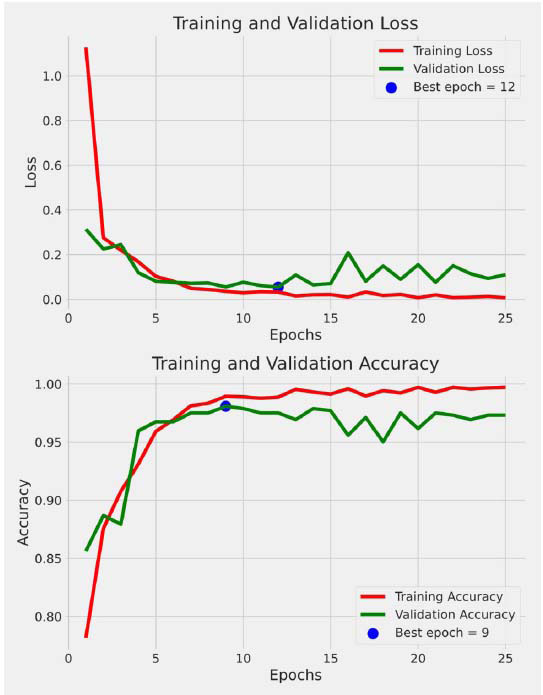
Graphical representation of training and validation loss and accuracy for PneumoNet.

Visualization plays a vital role in the inference of insight and visualization of the performance of PneumoNet. (Figure 7) shows training loss and accuracy versus validation loss and accuracy, along with the epochs, representing PneumoNet's learning efficiency and convergence habits.

## 4 EXPERIMENT AND ANALYSIS

The PneumoNet model was trained for 25 epochs using the Adamax optimizer, which was selected because of its ability to work with sparse gradients and perform well with noisy data. The learning rate was initially set at 0.001, in addition to the use of a learning rate scheduler that dynamically decreases the rate to assist in achieving better convergence. Early stopping with validation loss was used to prevent overfitting, enabling the model to function optimally without overtraining.

### 4.1 Results

The PneumoNet model demonstrated excellent accuracy on all datasets, attaining a training accuracy of 98.2%, a validation accuracy of 97.5%, and a test accuracy of 98.0%.

Table **2** presents a transparent and understandable view of the performance of the model on three datasets, which reflects well its consistency and strength. Precision and PneumoNet's accuracy are vital in the clinical setting in which the expense of false positives is very prohibitive with possible unwarranted treatments. The high precision and recall achieved by PneumoNet hold substantial practical value for healthcare practitioners. High precision is low false positives in the model, which is critical in medicine to avoid improper treatment or interventions based on an inaccurate diagnosis of pneumonia. Similarly, high recall enables the model to detect most true pneumonia cases, minimizing false negatives that could lead to diagnostic failures and suboptimal patient care.

The high Area Under the Curve (AUC) value of 0.99 in most datasets indicates that PneumoNet clearly separates the presence and absence of pneumonia. In real life, a high AUC value guarantees that the model has an incredibly high probability of correctly distinguishing between pneumonia and non-pneumonia X-rays, making it justifiable to use it as a diagnostic tool and be efficient. This skill is most beneficial in a high-case-load environment where immediate and accurate diagnosis is imperative to the successful and efficient implementation of treatment regimes.

Table **3** presents detailed information concerning every performance criterion for the PneumoNet model, satisfactorily highlighting its effectiveness and efficiency in chest X-ray image processing and pneumonia classification.

Fig. (**8**) illustrates that the confusion matrix gives an entire analysis of the performance of the model. It identified 980 pneumonia cases and 950 normal cases correctly, with 20 false positives and 30 false negatives. The matrix indicates that the model has high accuracy and low misclassification rates.

Fig. (**9**) presents a summary of the classification report for PneumoNet.

Fig. (**10**) shows cases the ROC curve for PneumoNet, displaying its discriminative power between the normal and pneumonia classes, along with the associated AUC score.

In comparison to ensemble techniques that fuse multiple models, PneumoNet showed better performance, mainly due to its optimized architecture and fine-tuned training process. The use of transfer learning also enhanced the generalizability and effectiveness of the model across datasets, highlighting the advantages of utilizing pre-trained models. Table **4** presents a comparison of performance metrics between PneumoNet and other prevalent models, with its higher accuracy and reliability in identifying pneumonia.

PneumoNet surpasses current state-of-the-art models in detecting pneumonia from chest X-ray images with robust performance metrics: a training accuracy of 98.2%, validation accuracy of 97.5%, and test accuracy of 98.0%. It exceeds other famous models such as MobileNet (accurate at 94.23%) and Deep Convolutional Neural Networks (accurate at 90.71%) from Enes Ayan *et al.* An AUC value of 0.99 for PneumoNet signifies it to be of superior discriminative power, demonstrating its functionality for clinical utility and technological ingenuity for usage.

Its success is attributed to the implementation of cutting-edge data augmentation strategies, efficient training practices, and an intricate CNN design. ROC-AUC scores and confusion matrix further reflect the model's discriminative power as well as its rate of misclassification, highlighting its precision in performance. Baseline comparisons with existing approaches are made to reflect the exceptional advantages of PneumoNet, particularly generalizability, and performance over different datasets. Translation into clinical applications in real-world settings is important and necessitates further testing and validation to address issues such as integration with existing healthcare systems and user adoption.

### 4.2 Ablation Study

An ablation experiment was conducted to examine the impact of different components of the PneumoNet architecture on the model's performance. Batch normalization being removed reduced accuracy to 95%, showcasing its capacity to stabilize learning. Removing dropout led to overfitting, with a decrease in validation accuracy to 93%, which justifies its requirement for model generalization. Reducing the model depth from five convolutional layers to three reduced accuracy to 96%, which suggests that network depth is critical in extracting complex features to yield accurate classifications. Increasing larger filter sizes reduced performance by a minimal amount to 97%, which suggests that small filters are optimal for fine-grained feature extraction in chest X-ray images. Replacing ReLU with sigmoid activation functions had another decrease in accuracy to 94%, which confirmed that ReLU is better in the category of maintaining the model effectively non-linear. The results emphasize how critical each of the provided modules is in maintaining high diagnostic accuracy and provide implications on what parts to further develop the PneumoNet model.

### 4.3 Discussion

This work presented PneumoNet, a new deep-learning network engineered particularly for pneumonia detection with maximum efficiency on chest X-rays, focusing on the architectural specifics and the evidence of its outperformance in diagnosis accuracy. The innovative organization of the convolutional layers in the model, along with well-advised applications of dropout and batch normalization, provides a solution to intrinsic medical image analysis issues, including excessive false positives and the ability to generalize across varied clinical environments. The ablation study not only justified the architecture choices but also left doors open for future optimization, where investigating hybrid convolutional filter sizes and more sophisticated regularization methods can possibly bring additional performance gains. In the future, the possibility of extending PneumoNet to the multi-disease detection scenario and into real-time clinical practice is a huge step in computational healthcare. This progress not only has the potential to increase the precision of diagnosis but also to relieve the medical community of much drudgery, opening the doors for wider applications of machine learning in medicine.

Possible shortcomings and areas of difficulty thereon must be addressed as we continue to implement it in true settings. One of the closest to primus is a class imbalance, the most common state among medical data sets where the presence of cases for normal states outnumber pneumonia or examples of any other pathology ten- or sometimes hundreds-to-one. This imbalance may cause models to be biased in favor of predicting the majority class, with the consequence of increased false negative rates for less common classes. Lack of consistency in imaging practices, *e.g*., variation in X-ray machine models, exposure levels, and patient positioning, may impact the performance of the model by causing inconsistencies in image quality and appearance. These inconsistencies can push the model's generalization capability to its limits on new, unseen data in other clinical environments.

Interpretability is still a major challenge in the use of deep learning models in medicine. The 'black box' feature of these models complicates understanding why particular diagnostic decisions are being made, and this is a major setback in clinical environments where explainability is needed to substantiate medical choices. It is dependent on enhancing their transparency and explainability to ask medical experts to trust and implement such models as PneumoNet. To scale these obstacles will require ongoing research and model building, for instance, techniques for balancing class distributions, more robustness to imaging practice variations, and the establishment of mechanisms to segregate and explain the model's decision-making.

## CONCLUSION

PneumoNet is a huge leap in the use of computer-aided diagnostic techniques in the healthcare industry, specifically in the diagnosis of pneumonia from X-ray chest images. By deploying advanced computational approaches and a high-performance CNN design, PneumoNet achieves not only precise diagnostic quality but also alleviates problems such as class imbalance and the enormous demand for annotated images. The model performs well with a training accuracy of 98.2%, validation accuracy of 97.5%, and test accuracy of 98.0%. Expansion of the dataset and refinement of the model architecture are future research agendas to further enhance performance and facilitate smoother integration into clinical workflows. Second, there is an attempt to broaden the potential of PneumoNet such that it will be used to diagnose other diseases, potentially being a more viable medical diagnostic instrument. Broadening its disease-detection capability comes with the necessity for rigorous validation to remain accurate in multiple pathologies.

Further advancement of PneumoNet will reduce the diagnostic load on health systems and significantly enhance patient care by increasing the accuracy and reliability of pneumonia detection. Regulatory hurdles need to be overcome to be effectively used in clinical settings, and worldwide health differences need to be addressed. The most important future directions are to avoid regulatory hurdles and facilitate the integration of PneumoNet into real-time clinical decision-making pathways. Besides, it is also necessary to address potential biases and the flexibility of the model in various settings to enable it to function optimally and ethically in real-world applications. This success marks a significant milestone in employing deep learning in supporting and supplementing clinical decision-making in real-time clinical practice.

## Figures and Tables

**Fig. (1a-c):**
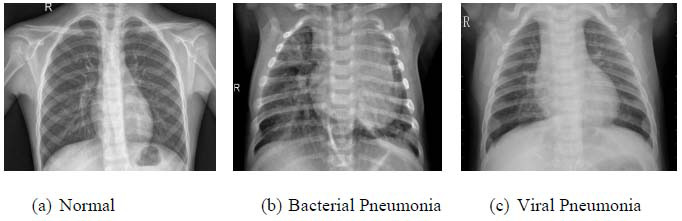
Chest X-ray images of pneumonia patients.

**Fig. (2):**
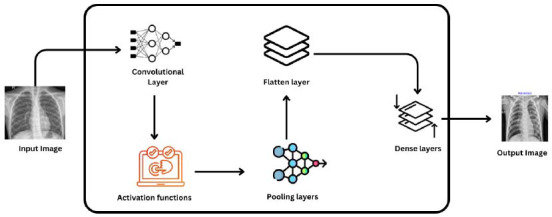
Workflow diagram of the Pneumonet model.

**Fig. (3):**
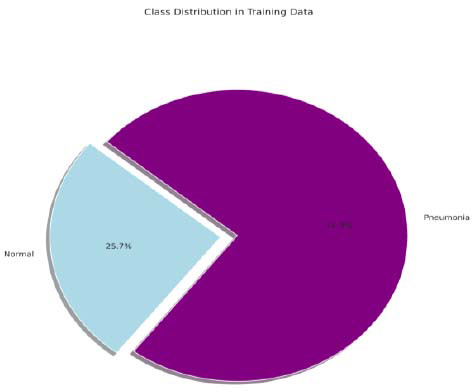
Class distribution in training data.

**Fig. (4a-p):**
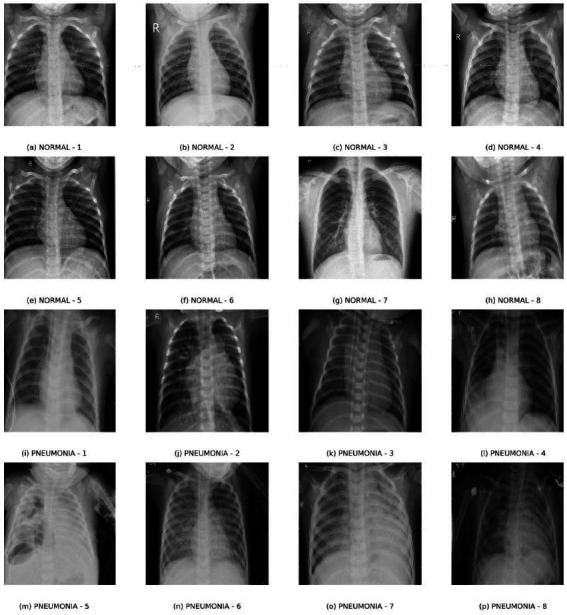
Sample input images from the “Chest X-ray images (pneumonia)” dataset.

**Fig. (5a-p):**
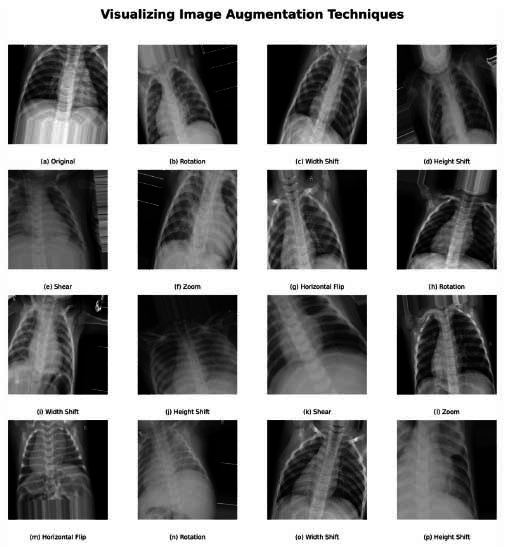
Visualization of augmented images.

**Fig. (6):**
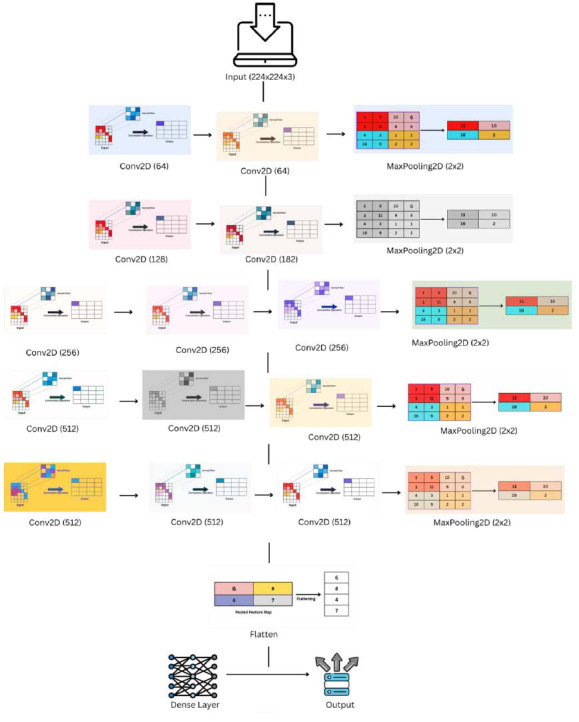
Architecture diagram.

**Fig. (8):**
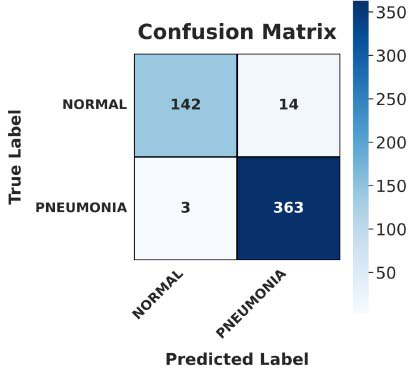
Confusion matrix of the PneumoNet.

**Fig. (9):**
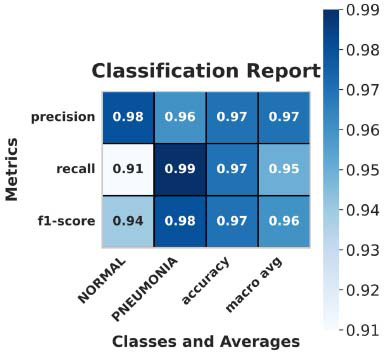
Classification report of the PneumoNet.

**Fig. (10):**
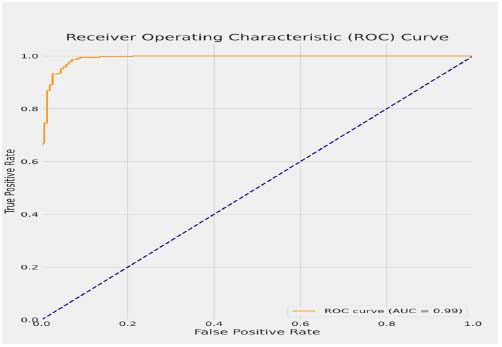
Roc curve for the analysis of PneumoNet.

**Table 1:** Review of existing literature and studies.

**Authors**	**Objective**	**Remarks**
Kundu *et al.* (2021) [5]	Develop a system for automatic pneumonia detection using chest X-ray images.	Demonstrated superior accuracy and sensitivity compared to current methods on public pneumonia X-ray datasets.
Goyal *et al.* (2021) [6]	Propose a framework for predicting lung diseases from chest X-ray images.	Showed robustness and efficiency, outperforming current methods in detecting lung diseases.
Račić *et al.* (2021) [7]	Build a CNN-based model for pneumonia detection from chest X-ray images.	Focused on enhancing diagnostic accuracy and consistency using AI and machine learning techniques.
Lenny *et al.* (2022) [8]	Enhance the accuracy of pneumonia diagnosis using deep learning models.	Compared different CNN architectures, providing insights for future advancements in pneumonia detection.
Li *et al.* (2022) [9]	Build a deep learning model for automated pneumonia detection using TensorFlow and Keras.	Enhanced speed and consistency of pneumonia diagnosis in clinical settings.
Guo *et al.* (2023) [10]	Develop and evaluate a weakly supervised algorithm for pneumonia detection using X-ray images.	ResNet34 model demonstrated high accuracy and sensitivity, supporting radiologists in detecting and locating pneumonia lesions.
Wardah *et al.* (2023) [11]	Address class imbalance in pneumonia detection using GAN-based data augmentation.	Superior performance in handling class imbalance and improved accuracy with transfer learning on deep learning models.
Akbulut *et al.* (2023) [12]	Propose a customized deep-learning model for classifying lung diseases.	ACL model showed high accuracy across training-test ratios, proving its reliability for early diagnosis of lung diseases.
Sharma *et al.* (2024) [13]	Review various deep-learning techniques for detecting pneumonia.	Provided comprehensive insights into the performance, architecture, and effectiveness of deep learning models.
Ali *et al.* (2024) [14]	Evaluate six deep learning models, including EfficientNetV2L, for pneumonia detection.	EfficientNetV2L achieved the highest accuracy, demonstrating robustness and efficacy in clinical settings.
Ravi *et al.* (2024) [15]	Develop a hybrid deep-learning model for pediatric pneumonia detection.	Enhanced detection accuracy by 4% over existing methods, showing robust performance on unseen datasets and similar diseases.

**Table 2:** Summary of performance metrics for PneumoNet.

**Metric**	**Training Set**	**Validation Set**	**Test Set**
Accuracy	98.2%	97.5%	98.0%
Precision	96.0%	95.8%	98.0%
Recall	96.0%	95.9%	98.0%
F1-score	96.0%	95.8%	98.0%
AUC score	0.99	0.98	0.99

**Table 3:** Computation time and performance metrics for PneumoNet.

**Metric**	**Value**
Model name	PneumoNet
Accuracy	98.0%
Precision	98.0%
Recall	98.0%
F1-score	98.0%
AUC score	0.99
Computation time (s)	120

**Table 4:** Comparative performance of pneumonet and existing models.

Authors	Techniques	Accuracy
Jianpeng Zhang *et al.* (2021) [22]	Confidence-aware anomaly detection (CAAD) model	78.52%
Alhassan Mabrouk *et al.* (2022) [23]	Ensemble of Deep Convolutional Neural Networks	93.91%
Enes Ayan *et al.* (2022) [24]	Deep Convolutional Neural Networks	90.71%
César Ortiz-Toro *et al.* (2022) [25]	Automatic machine learning techniques (KNN, SVM, and RF)	95%
Shagun Sharma *et al.* (2023) [26]	VGG16 with Neural Networks (NN)	95.4%
Harsh Bhatt *et al.* (2023) [27]	Convolutional Neural Network ensemble model	85.58%
Mana Saleh Al Reshan *et al.* (2023) [28]	MobileNet Model	94.23%
Sukhendra Singh *et al.* (2023) [29]	QCSA network (Quaternion Channel-Spatial Attention Network)	94.53%
Md. Maniruzzaman *et al.* (2024) [30]	Deep learning models such as VGG-16, VGG-19, ResNet-50, Inception-V3, Xception pre-trained on ImageNet	95.06%
Maquen-Niño *et al.* (2024) [31]	Transfer Learning	91%
Proposed Model	Deep Learning Model	98%

## Data Availability

The dataset is publicly available on Kaggle (https://www.kaggle.com/datasets/paultimothymooney/chest-xray-pneumonia).

## LIST OF ABBREVIATIONS

CNN: Convolutional Neural Network
ReLU: Rectified Linear Unit
AUC: Area Under the Curve
